# Correlation of anemia severity with maternal cardiac function during third trimester - An observational study

**DOI:** 10.6026/973206300223277

**Published:** 2026-06-30

**Authors:** Sarita Jalodiya, Nidhi Narrey, Sarita Shah

**Affiliations:** 1 Department of Medicine, NSC Government Medical College, Khandwa, Madhya Pradesh, India; 2 Department of Obstetrics and Gynecology, Chhindwara Institute of Medical Sciences, Chhindwara, Madhya Pradesh, India; 3 Department of Obstetrics and Gynecology, Government Medical College, Singrauli, Madhya Pradesh, India

**Keywords:** Maternal anemia, pregnancy, cardiac function, echocardiography, third trimester, hemoglobin, maternal cardiovascular adaptation

## Abstract

Maternal anemia is a common pregnancy condition that can considerably impair cardiovascular adaption throughout the third trimester. Therefore, it is interest to report. The relationship between anemia severity and maternal cardiac function in third-trimester pregnant women using echocardiographic measures. Hence, a total of 120 pregnant women were divided into mild, moderate, and severe anemia groups based on hemoglobin levels, and cardiac function parameters such heart rate, cardiac output, and left ventricular ejection fraction were measured. An increasing severity of anemia was associated with considerably greater heart rate and cardiac output, as well as minor impairments in diastolic cardiac function. Thus, we report that increasing severity of maternal anemia in the third trimester produces measurable compensatory cardiac changes rising heart rate and cardiac output with early diastolic impairment underscoring the need for routine echocardiographic surveillance in anemic pregnant women.

## Background:

Anemia during pregnancy is still one of the most common nutritional problems globally, affecting roughly 40% of pregnant women and contributing considerably to maternal and fetal morbidity. The illness is most prevalent in developing nations due to iron shortage, dietary insufficiency, and higher physiological demands during pregnancy [1]. Maternal anemia has been linked to unfavorable pregnancy outcomes such as preterm birth, low birth weight, and intrauterine growth restriction [2]. Significant physiological cardiovascular changes occur throughout pregnancy in order to meet the metabolic demands of both mother and fetus. These alterations include higher plasma volume, increased cardiac output, lower systemic vascular resistance, and faster heart rate. When anemia occurs, the oxygen-carrying capacity of the blood declines, prompting compensatory cardiovascular responses such as tachycardia and increased stroke volume to maintain adequate tissue oxygenation [3]. Previous research has shown that anemia can affect myocardial workload and cause structural and functional cardiac abnormalities during pregnancy. Echocardiography has long been used as a non-invasive method to assess cardiac function and monitor hemodynamic changes in pregnant women [4]. Recent research suggests that iron deficiency anemia during pregnancy can produce subtle cardiac functional alterations that can be detected using advanced echocardiographic markers such as myocardial strain [5]. Therefore, it is of interest to examine the link between the severity of anemia and maternal cardiac function using echocardiographic measures in third-trimester pregnant women.

## Methods:

This observational cross-sectional study was carried out between January 2024 and December 2025 in a tertiary care teaching hospital's Department of Obstetrics and Gynecology. The study comprised 120 pregnant women in their third trimester (28-40 weeks) who were attending an antenatal clinic. The participants were divided into three groups based on WHO hemoglobin criteria: mild anemia (10-10.9 g/dL; n=40), moderate anemia (7-9.9 g/dL; n=40), and severe anemia (<7 g/dL). Hemoglobin levels were determined using an automated hematology analyzer.

## Inclusion criteria:

[1] Pregnant women aged 18-40 years

[2] Singleton pregnancy

[3] Gestational age ≥28 weeks

[4] Diagnosed anemia according to WHO criteria

[5] Willingness to participate and provide informed consent

## Exclusion criteria:

[1] Pre-existing heart disease

[2] Hypertensive disorders of pregnancy

[3] Diabetes mellitus

[4] Multiple pregnancy

[5] Thyroid disorders

[6] Chronic renal disease

[7] Known congenital fetal anomalies

## Echocardiographic evaluation:

All participants underwent two-dimensional transthoracic echocardiography performed by a cardiologist using standard guidelines. The following parameters were measured:

[1] Heart rate (HR)

[2] Left ventricular ejection fraction (LVEF)

[3] Stroke volume (SV)

[4] Cardiac output (CO)

E/A ratio (diastolic function indicator) 

## Statistical analysis:

All data was analyzed with the Statistical Package for Social Sciences (SPSS) version 26.0. Continuous data including were reported as mean ± SD, whereas categorical variables were presented as frequency and %. Continuous variables were compared using one-way analysis of variance (ANOVA), with post-hoc analysis as needed. The Pearson correlation coefficient was used to assess the association between hemoglobin levels and echocardiographic parameters. A p-value of <0.05 was judged statistically significant.

## Results:

A total of 120 pregnant women were analyzed in the present study during the third trimester. The study participants were categorized according to the severity of anemia into mild anemia (n=40), moderate anemia (n=40), and severe anemia (n=40) groups. The demographic characteristics such as age, gestational age, and BMI were comparable among mild, moderate, and severe anemia groups, with no statistically significant differences (p>0.05) (Table 1). Hemoglobin and hematocrit levels significantly decreased with increasing severity of anemia across the three groups (p<0.001) (Table 2). Heart rate, stroke volume, and cardiac output showed a significant increase as the severity of anemia increased (p<0.001) (Table 3). Left ventricular ejection fraction and E/A ratio showed a gradual decline with increasing anemia severity, indicating early cardiac functional changes (Table 4). The Figure 1 (see PDF) shows an inverse relationship between hemoglobin level and maternal cardiac output, with higher cardiac output observed in severe anemia. The Figure 2 (see PDF) shows a progressive decrease in E/A ratio from mild to severe anemia, suggesting worsening diastolic cardiac function the graphical representation (Figure 1 - see PDF) shows an inverse relationship between hemoglobin level and maternal cardiac output, indicating increased cardiac output with increasing severity of anemia. The graphical representation (Figure 2 - see PDF) shows a gradual decrease in E/A ratio with increasing severity of anemia, suggesting early impairment of diastolic cardiac function.

## Discussion:

Maternal anemia has a major impact on cardiovascular physiology during pregnancy because it reduces oxygen-carrying capacity and increases metabolic demand. In the present investigation, we noticed a considerable rise in heart rate and cardiac output as the severity of anemia increased, indicating compensatory cardiovascular mechanisms. Previous research has found that anemia causes increased cardiac output and decreased systemic vascular resistance in order to keep oxygen flowing to maternal tissues and the fetus [6]. The increased burden on the heart may predispose women suffering from severe anemia to cardiac stress and malfunction. Our findings also revealed minor reductions in left ventricular ejection fraction and E/A ratio in women with severe anemia, indicating early deterioration in both systolic and diastolic cardiac function. Recent echocardiographic studies have revealed that iron deficiency anemia during pregnancy might cause subclinical myocardial functional alterations, even if the ejection fraction stays normal [7]. Echocardiography has emerged as a useful noninvasive method for assessing circulatory changes throughout pregnancy and detecting early heart disease. According to recent research, echocardiographic surveillance may be effective in women with severe anemia for detecting early cardiovascular impairment [8]. Furthermore, anemia during pregnancy has been linked not only to maternal cardiovascular strain but also to poor maternal and fetal outcomes, stressing the importance of early detection and proper treatment [1, 2]. The findings of our study highlight the necessity of frequent screening and timely treatment of anemia during pregnancy. However, the current study has several limitations, including a limited sample size and a single-center design. Larger multicenter trials with longitudinal follow-up might help to better understand the long-term cardiovascular consequences of maternal anemia.

## Conclusion:

The severity of maternal anemia during the third trimester is highly related with changes in maternal cardiac function. As the severity of anemia increases, so does the heart rate, cardiac output, and early echocardiographic alterations, indicating cardiovascular adaptation and strain. Thus, data shows the necessity of early detection and treatment of anemia during pregnancy in order to avoid potential maternal cardiovascular problems. Routine echocardiographic evaluation in severe anemia may aid in the early diagnosis of heart dysfunction and improve maternal outcomes.

## Figures and Tables

**Table 1:** Demographic characteristics

**Parameter**	**Mild anemia**	**Moderate anemia**	**Severe anemia**	**p value**
Age (years)	25.4 ± 3.6	26.1 ± 4.1	25.8 ± 3.9	0.74
Gestational age (weeks)	33.2 ± 2.4	33.6 ± 2.1	33.4 ± 2.3	0.82
BMI (kg/m^2^)	24.3 ± 2.5	24.9 ± 2.2	24.7 ± 2.6	0.65

**Table 2:** Hematological parameters

**Parameter**	**Mild**	**Moderate**	**Severe**	**p value**
Hemoglobin (g/dL)	10.4 ± 0.3	8.6 ± 0.7	6.4 ± 0.5	<0.001
-Hematocrit (%)	32.2 ± 2.1	28.3 ± 2.4	23.5 ± 1.8	<0.001

**Table 3:** Maternal cardiac function parameters

**Parameter**	**Mild**	**Moderate**	**Severe**	**p value**
Heart Rate (bpm)	82 ± 6	88 ± 7	96 ± 8	<0.001
Stroke Volume (ml)	72 ± 8	78 ± 7	84 ± 6	<0.001
Cardiac Output (L/min)	5.9 ± 0.6	6.8 ± 0.7	7.9 ± 0.9	<0.001

**Table 4:** Echocardiographic parameters

**Parameter**	**Mild**	**Moderate**	**Severe**	**p value**
LVEF (%)	63.4 ±4.2	61.9 ±4.5	59.7 ±4.8	0.03
E/A ratio	1.42 ±0.2	1.34 ±0.2	1.20 ±0.3	0.01
